# The Hippo pathway promotes platinum-based chemotherapy by inhibiting MTF1-dependent heavy metal response

**DOI:** 10.1186/s12885-025-13661-8

**Published:** 2025-02-08

**Authors:** Hui Chen, Yue Xu, Dingshan Chen, Di Xiao, Bing Yang, Wenqi Wang, Han Han

**Affiliations:** 1 Department of Pathophysiology, TaiKang Center for Life and Medical Sciences, TaiKang Medical School (School of Basic Medical Sciences), Wuhan University, Wuhan, 430071 Hubei China; 2 Department of Developmental and Cell Biology, University of California, Irvine, CA 92697 USA

**Keywords:** Platinum, Chemotherapy, Cisplatin, Hippo pathway, MTF1

## Abstract

The platinum-based compounds are widely used in treating various types of cancer through their heavy metal component platinum. However, the development of chemoresistance often limits their clinical effectiveness. In this study, we report the roles of heavy metal response and its associated Hippo pathway in regulating platinum-based chemotherapy. Our data show that the MTF1-dependent heavy metal response induces cancer cell resistance to platinum-based compounds both in vitro and in vivo. This resistance is mitigated by Hippo pathway-mediated phosphorylation of MTF1. Moreover, pharmacological activation of the Hippo pathway sensitizes cancer cells to platinum-based compounds. Clinically, lung adenocarcinoma (LUAD) patients with high MTF1 activity exhibit poor overall survival rates, and Hippo pathway inactivation is positively correlated with elevated MTF1 transcriptional activity in platinum-treated LUAD patients. Collectively, our findings not only unveil a critical role of the Hippo-MTF1 pathway in regulating the response to platinum-based chemotherapy, but also suggest new strategies to enhance its efficacy by targeting the heavy metal response.

**Supplementary Information:**

The online version contains supplementary material available at 10.1186/s12885-025-13661-8.

## Background

Platinum-based compounds are potent chemotherapy agents extensively used to treat various cancers, including ovarian cancer, endometrial cancer, head and neck cancer, lung cancer, breast cancer [1–3]. These drugs work by reacting with purine bases on DNA, leading to DNA crosslinking, strand breaks, impaired cell division, and cell death. However, cancer cells often develop resistance to platinum-based therapies due to alterations in DNA repair mechanisms and anti-apoptotic pathways, resulting in poor therapeutic outcomes [4–6].

Notably, platinum-based compounds are unique among chemotherapy drugs as they contain heavy metal platinum. Upon exposures to heavy metals, metal regulatory transcription factor 1 (MTF1) is translocated from the cytoplasm into the nucleus, where it binds to metal response element (MRE) in the promoter/enhancer regions of heavy metal response genes, inducing their expression [7, 8]. Among them, metallothioneins (MTs) are single-chain polypeptides rich in cysteine residues that form unique structures to bind metals via sulfur atoms in thiolate clusters, thereby protecting cells from heavy metal-induced toxicity [9–12]. In addition, the expression of metal transporters is upregulated to facilitate the efflux of excessed heavy metals [8]. However, whether platinum-based chemotherapy drugs induce heavy metal response similar to other heavy metals and how this response affects platinum-based chemotherapy are key questions that are not yet fully understood.

As an evolutionarily conserved pathway, the Hippo pathway plays a crucial role in development, tissue homeostasis, organ size control, and cancer development [13–18]. In mammals, the core kinase cascade of the Hippo pathway consists of two Ser/Thr kinases LATS and MST along with their adaptors MOB1 and SAV1, respectively. MST phosphorylates and activates LATS, which in turn phosphorylates two transcriptional co-activators YAP and TAZ, leading to their cytoplasmic retention by 14–3-3 and followed proteasomal degradation [15, 16, 19]. When the Hippo pathway is inactive, unphosphorylated YAP and TAZ enter the nucleus, where they form a complex with the transcription factor TEAD to drive the expression of genes involved in a variety of growth-related events [20–22]. NF2, a membrane-localized protein, is an upstream component of the Hippo pathway required for LATS kinase activation [23, 24]. Our recent work revealed that the Hippo pathway acts as a negative regulator of the heavy metal response through LATS-mediated phosphorylation of MTF1 at S152, thereby inhibiting its DNA binding and downstream heavy metal gene transcription, a process independent of YAP and TAZ [25, 26]. However, the role of the Hippo-MTF1 pathway in the platinum-based chemotherapy and its potential involvement in chemoresistance remain poorly understood.

In this study, we report the critical role of the Hippo-MTF1 pathway in regulating platinum-based chemotherapy. Our data demonstrate that platinum-based drugs activate MTF1 and induce the expression of downstream heavy metal response genes in various cancer cell lines. Loss of MTF1 significantly reduces cell viability and tumor growth in response to platinum-based drugs cisplatin, and this inhibition can be reversed by expressing heavy metal response genes encoding for MT1A and MT2A. In addition, targeting either the Hippo pathway or LATS-induced MTF1 S152 phosphorylation protected cancer cells from cisplatin treatment, while pharmacological activation of the Hippo pathway enhances cisplatin sensitivity. Clinically, lung adenocarcinoma (LUAD) patients with high levels of heavy metal response genes exhibit poor overall survival rates, and heavy metal response gene expression is positively correlated with Hippo signaling downstream genes in tumor tissues from LUAD patients following platinum drug treatment. Taken together, our findings reveal the Hippo-MTF1 pathway as a key regulator of platinum-based chemotherapy, offering novel insights into mechanisms of chemoresistance and potential sensitizing strategies.

## Materials and methods

### Cell lines

HEK293T (a female cell line, ATCC: CRL-3216) was purchased from ATCC and kindly provided by Dr. Junjie Chen (MD Anderson Cancer Center). HEK293A (a female cell line, Thermo Fisher Scientific: R70507) cells were kindly provided by Dr. Jae-Il Park (MD Anderson Cancer Center). HEY (a female cell line, ATCC: CRL-3252), HEC1A (a female cell line, ATCC: HTB-112), and CAL-27 (a male cell line, ATCC: CRL-2095) cell lines were purchased from ATCC. HEK293T, HEK293A and CAL-27 cells were maintained in Dulbecco's Modified Eagle's Medium (DMEM). HEY cells were maintained in RPMI-1640 Medium. HEC1A cells were maintained in McCoy's 5A Medium. All the culture media were supplemented with 10% fetal bovine serum and contain 1% penicillin and streptomycin. All the cells were maintained at 37 °C in 5% CO2 (v/v).

### Antibodies and chemicals

For Western blotting, anti-MTF1 (NBP1-86,380, 1:2000 dilution) antibody was obtained from Novus Biologicals. Anti-Flag (M2)-peroxidase (HRP) (A8592-1MG, 1:5000 dilution) and anti-α-tubulin (T6199-200UL, 1:5000 dilution) antibodies were obtained from Sigma-Aldrich. Anti-phospho-LATS1 (Thr1079) (8654S, 1:1000 dilution) and anti-LATS1 (3477S, 1:1000 dilution) antibodies were purchased from Cell Signaling Technology. For immunofluorescent staining, anti-MTF1 antibody (NBP1-86,380, 1:200 dilution) was obtained from Novus Biologicals.

Cisplatin (AG-CR1-3590) was purchased from Adipogen. Carboplatin (C2538), oxaliplatin (O9512), FIPI (F5870), cerivastatin (SML0005), and CdCl2 (655,198) were purchased from Sigma-Aldrich. Mitomycin C (HY-13316) and camptothecin (HY-16560) were obtained from Med Chem Express.

### Constructs and viruses

Plasmids encoding the indicated genes were obtained from the Human ORFeome V5.1 library or purchased from DNASU Plasmid Repository. The constructs were generated via polymerase chain reaction (PCR) and sub-cloned into a pDONOR201 vector using Gateway Technology (Thermo Fisher Scientific) as entry plasmids. A lentiviral gateway-compatible destination vector with the SFB tag was used to express various fusion proteins. PCR-based mutagenesis was used to generate the indicated site mutations.

Lentiviral supernatants were generated by transient transfection of HEK293T cells with the helper plasmids pSPAX2 and pMD2G and harvested 48 h later. Supernatants were passed through a 0.45-µm filter and used to infect cells with the addition of 8 µg/mL hexadimethrine bromide (Polybrene) (Sigma-Aldrich). Plasmid transfection was performed using polyethyleneimine (PEI) (23,966–2, Polysciences).

### Bioinformatic analysis

Clinical information of genes *CTGF, CYR61, MT1A, MT2A, YAP1, WWTR1* and *MTF1* was obtained from The Cancer Genome Atlas (TCGA) data portal (http://tcga-data.nci.nih.gov). mRNA expression level of the indicated genes was analyzed in lung cancer (TCGA-LUAD) and head and neck cancer (TCGA-HNSC) using GraphPad spearman correlation. Each point indicates an individual tissue sample. Additionally, mRNA expression level of *MT1*-family genes and *MT2A* was analyzed in breast and ovarian cancers using Kaplan–Meier Plotter (https://kmplot.com/analysis/). R, correlation coefficient. As for the patient survival studies, the top one-third expression level of each gene among all patients was classified as high, while the remaining two-thirds were classified as low. Survival data for each group were analyzed by log-rank (Mantel-Cox) test.

### Generation of MTF1 knockout (KO) cell lines using CRISPR/Cas9

The indicated LATS1/2 double knockout (DKO), MOB1A/B DKO, NF2 KO, MTF1 KO, LATS1/2/MTF1 triple knockout (TKO) HEK293A cells were generated as described previously [25, 27]. The same single-guide RNAs (sgRNA) set was used to generate the MTF1 KO HEY, HEC1A and CAL-27 cells. Briefly, five sgRNAs of MTF1 were designed by CHOPCHOP website (https://chopchop.rc.fas.harvard.edu), cloned into the lentiGuide-Puro vector (Addgene plasmid # 52,963), and transfected into each cancer cell line with the lentiCas9-Blast construct (Addgene plasmid # 52,962). The next day, cells were selected with puromycin (2µg/ml) for two days and subcloned to form single colonies. Knockout cell clones were screened by Western blot to verify the loss of MTF1 expression. The genomic editing was further confirmed by sequencing.

### Immunofluorescent staining

Immunofluorescent staining was performed as described previously [28]. Briefly, cells cultured on coverslips were fixed with 4% paraformaldehyde for 10 min at room temperature and then extracted with 0.5% Triton X-100 solution for 5 min. After blocking with Tris-buffered saline with Tween 20 containing 1% bovine serum albumin, the cells were incubated with the indicated primary antibodies for 1 h at room temperature. After that, cells were washed and incubated with rhodamine-conjugated secondary antibodies for 1 h. To visualize nuclear DNA, cells were counterstained with 100 ng/mL 4′,6-diamidino-2-phenylindole (DAPI) for 2 min. The cover slips were mounted onto glass slides with an anti-fade solution and visualized under a Nikon Ti2-E inverted microscope.

### Cell viability assay

For crystal violet staining assay, cells were seeded in 12-well plates and subjected to the indicated treatments. At the endpoint, cells were fixed with 4% paraformaldehyde for 10 min and stained with 0.1% crystal violet. Cells were then washed for three times and detained with acetic acid. The absorbance of the crystal violet solution was measured at O.D. 595 nm and normalized to vehicle-treated cells.

### RNA extraction, reverse transcription, and real-time PCR

RNA samples were extracted with TRIzol reagent (Invitrogen). Reverse transcription assay was performed with the Script Reverse Transcription Supermix Kit (Bio-Rad) according to the manufacturer’s instruction. Real-time PCR was performed using Power SYBR Green PCR master mix (Applied Biosystems). For quantification of gene expression, the 2^−ΔΔCt^ method was used. *GAPDH* expression was used for normalization. The sequence information of q-PCR primers used for gene expression analysis is as follows:

- *MT1A*-Forward: 5′- CAGCTGCACTTCTCTGATGC-3′;
- *MT1A*-Reverse: 5′- AATGCAACTCCTGCAAGAAGA-3′;
- *MT1F*-Forward: 5′- TCTCTTGGAAAGTCCAGTCTC-3′;
- *MT1F*-Reverse: 5′- ACTCTTTGCACTTGCAGGA-3′;
- *GAPDH*-Forward: 5′-ATGGGGAAGGTGAAGGTCG-3′;
- *GAPDH*-Reverse: 5′-GGGGTCATTGATGGCAACAATA-3′.

### Xenograft tumor assays

All the xenograft tumor assays were followed with institutional guidelines, approved by the Institutional Animal Care and Use Committee (IACUC; protocol number AUP-19–112) of University of California, Irvine (UCI), and performed under veterinary supervision. Athymic nude (nu/nu) mouse strain was used for the xenograft tumor assays in this study. All the nude mice were purchased from Jackson Laboratory and kept in a pathogen-free environment at the UCI ULAR facility. The indicated HEY and HEC1A cells (2 × 10^6^) were subcutaneously injected into nude mice. When tumors were approximately 50 mm^3^ in size, 10 mice for each cell line were randomly assigned into two groups (5 mice per group) and subjected to cisplatin treatment (5 mg/kg) twice a week. After three weeks for treatment, mice were euthanized, and tumor weights were analyzed.

For subcutaneous injection, isoflurane anesthesia was used to release the pain of mice. Specifically, mice were anesthetized with an initial concentration of 4‐5% isoflurane mixed with oxygen in an induction chamber. When mice were unconscious, they were transferred to a nosecone to continue delivery of isoflurane in oxygen. Depth of anesthesia was evaluated by lack of response to toe pinch, touching of eyelids or other stimulus, and evaluation of the respiration rate. Anesthesia depth was re‐assessed at 2‐3 min intervals for the duration of the procedure. Consistent with the recommendations of the Panel on Euthanasia of the American Veterinary Medical Association, all animals were sacrificed by means of inhalation of carbon dioxide gas. Carbon dioxide was introduced into the cage at a 10 ~ 30% fill rate per minute until no movement of mice was noted for at least one minute. Mice were subsequently subjected to cervical dislocation as a secondary method to verify euthanasia.

### Quantification and statistical analysis

Patient survival data were analyzed by log-rank (i.e., Mantel-Cox) test. Gene correlation data were analyzed by spearman correlation. There were no samples or animals excluded from studies. There was no statistical method used to predetermine sample size for the mouse xenograft tumor experiments. The Student’s *t*-test (two-sided) was used to analyze the differences between two sample groups. SD was used for error estimation. Each experiment was repeated twice or more, unless otherwise noted. A *p* value < 0.05 was considered statistically significant.

## Results

### Platinum-based compounds activate MTF1

**Fig. 1:**
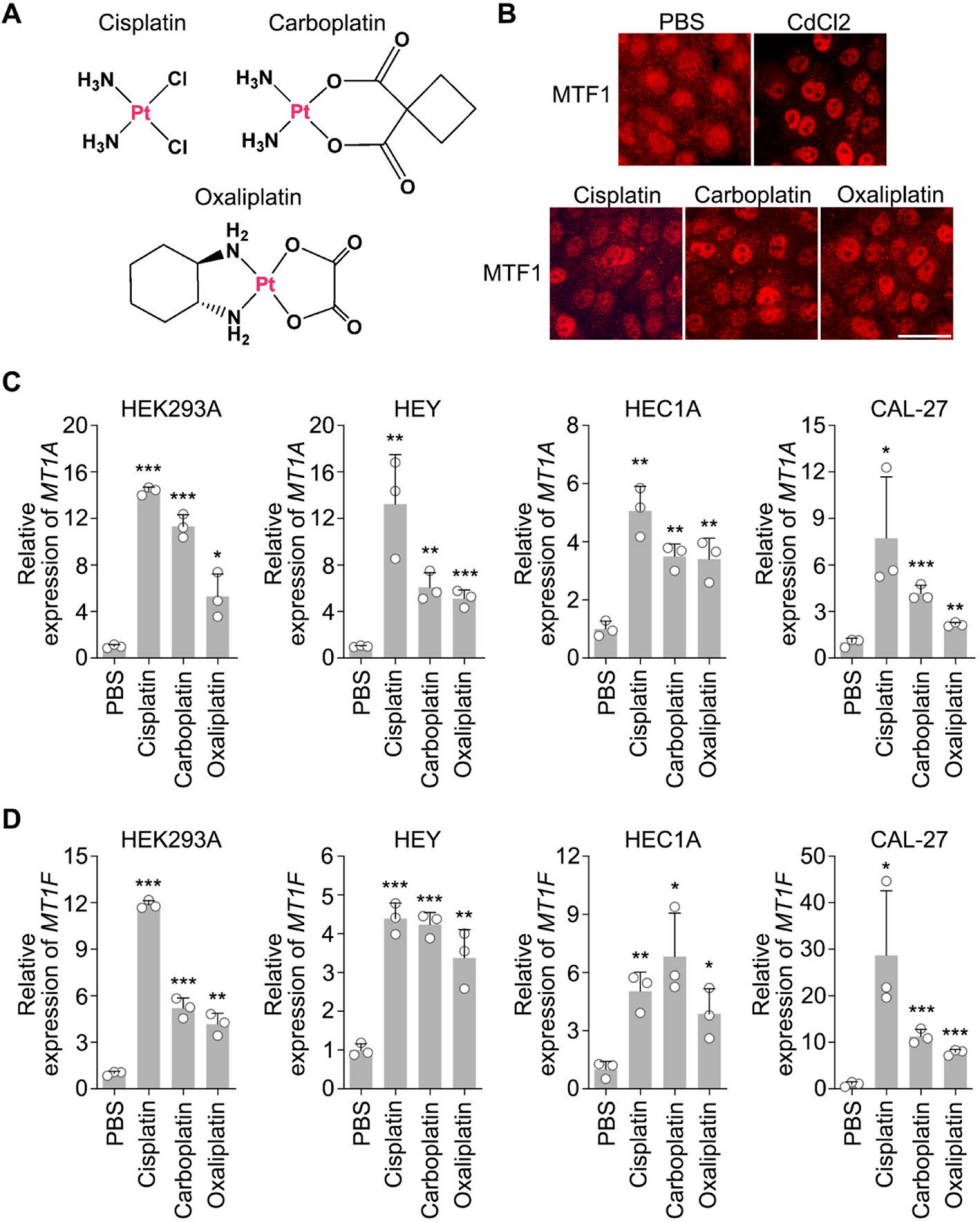
Platinum-based chemotherapy drugs activate MTF1 and the heavy metal response. **A** Illustration of the chemical structures for platinum-based compounds cisplatin, carboplatin and oxaliplatin. Platinum (Pt) is labeled in red. **B** Platinum-based compounds induce MTF1’s nuclear translocation. HEK293A cells were treated with CdCl2 (50 µM), cisplatin (50 µM), carboplatin (200 µM) and oxaliplatin (100 µM) for 4 h and subjected to immunofluorescent staining. Scale bar, 20 µm. **C-D** Platinum-based compounds promote the transcription of heavy metal response genes. The transcription of heavy metal response genes *MT1A* (**C**) and *MT1F* (**D**) was examined in the indicated cell lines by q-PCR (mean ± s.d., n = 3 biological replicates). The indicated cells were treated with cisplatin (50 µM), carboplatin (200 µM) and oxaliplatin (100 µM) for 12 h. * *p* < 0.05, ** *p* < 0.01, *** *p* < 0.001 (Student’s *t*-test)

To determine the role of platinum-based chemotherapy drugs in regulating the heavy metal response, we treated HEK293A cells with cisplatin, carboplatin and oxaliplatin (Fig. 1A). Like heavy metal cadmium (Cd), all these platinum-based drugs induced the nuclear translocation of MTF1 (Fig. 1B). Furthermore, treatment with platinum-based drugs significantly increased the transcription of MTF1 downstream genes *MT1A* (Fig. 1C) and *MT1F* (Fig. 1D) in different cell lines. These results suggest that platinum-based chemotherapy drugs activate MTF1 and the heavy metal response.

### Loss of MTF1 sensitizes cancer cells to cisplatin treatment

**Fig. 2:**
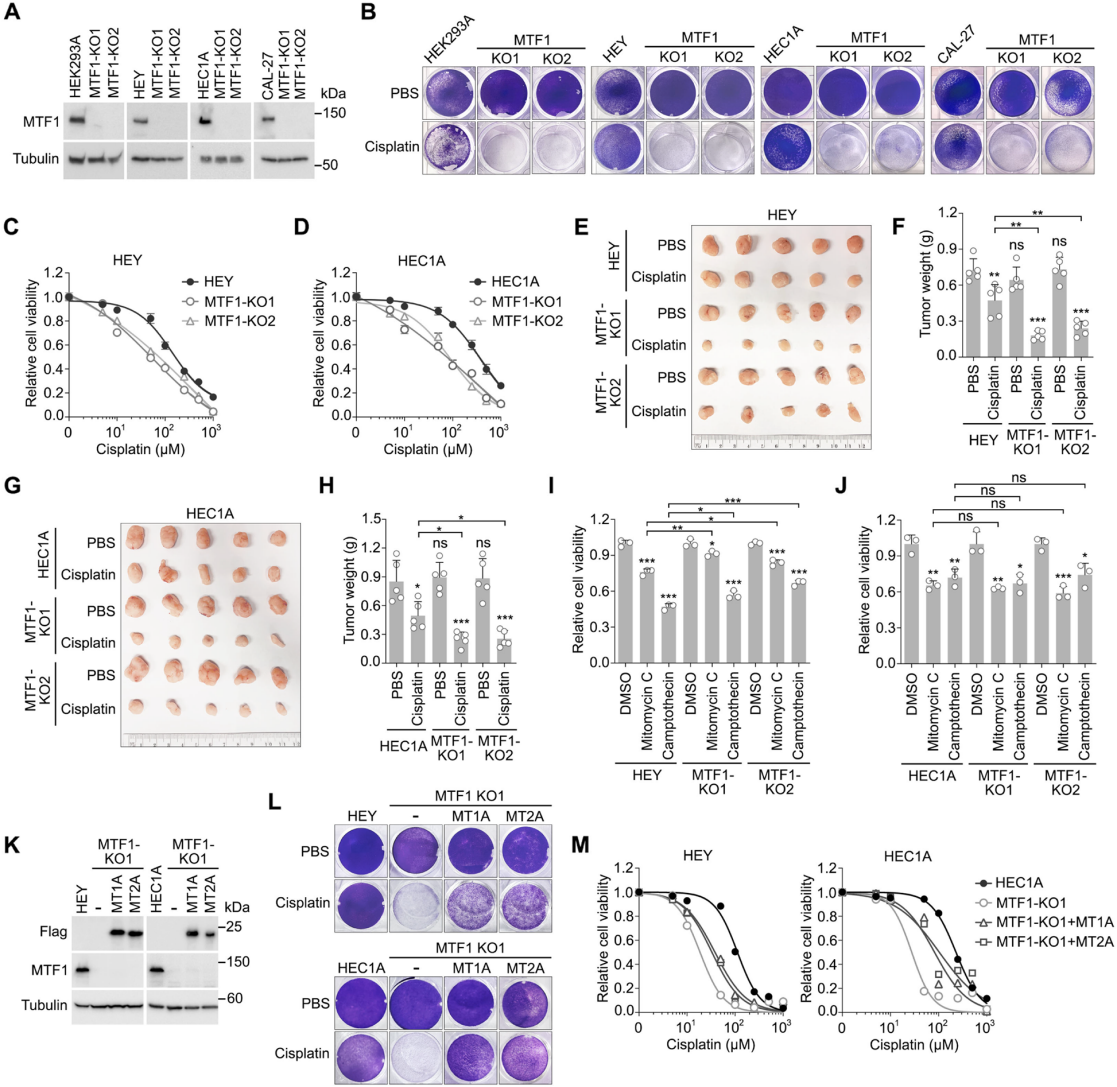
MTF1 and its associated heavy metal response induce resistance to platinum-based chemotherapy. **A** Validation of the MTF1 knockout (KO) cells by Western blot. **B-D** Loss of MTF1 inhibits cell viability under cisplatin treatment. The indicated wild-type and the MTF1 KO cells were treated with cisplatin (50 µM for HEK293A, 100 µM for HEY, 100 µM for HEC1A, and 10 µM for CAL-27) for 24 h and visualized by crystal violet staining (**B**). Wild-type and MTF1 KO HEY (**C**) and HEC1A (**D**) cells were treated with the indicated concentration of cisplatin for 24 h and quantified for relative viability (mean ± s.d., *n* = 3 biological replicates). **E–F** Loss of MTF1 inhibits tumor growth under cisplatin treatment. Wild-type and MTF1 KO HEY cells were subjected to xenograft study and treated with cisplatin (5 mg/kg) twice a week. The collected tumors at the endpoint were shown (**E**), and tumor weight was measured and quantified (mean ± s.d., *n* = 5 mice per group) (**F**). ** *p* < 0.01, *** *p* < 0.001 (Student’s *t*-test). ns, no significance. **G-H** Wild-type and MTF1 KO HEC1A cells were subjected to xenograft study and treated with cisplatin (5 mg/kg) twice a week. The collected tumors at the endpoint were shown (**E**), and tumor weight was measured and quantified (mean ± s.d., *n* = 5 mice per group) (**F**). * *p* < 0.05, *** *p* < 0.001 (Student’s *t*-test). ns, no significance. **I-J** Loss of MTF1 does not affect mitomycin C and camptothecin-induced cell death. Wild-type and MTF1 KO HEY (**I**) and HEC1A (**J**) cells were treated with mitomycin C (20 µM) and camptothecin (50 µM) for 24 h, subjected to crystal violet staining, and quantified for relative viability (mean ± s.d., *n* = 3 biological replicates). * *p* < 0.05, ** *p* < 0.01, *** *p* < 0.001 (Student’s *t*-test). ns, no significance. **K-M** Reconstitution of heavy metal response proteins MT1A and MT2A rescues MTF1 KO cell viability under cisplatin treatment. The indicated MTF1 KO cells were reconstituted with MT1A and MT2A (**K**), treated with cisplatin (10 µM for HEY, 25 µM for HEC1A) for 48 h, and visualized by crystal violet staining (**L**). Wild-type HEY and HEC1A and their indicated MTF1 KO cells were treated with the indicated concentration of cisplatin for 48 h and quantified for relative viability (mean, *n* = 3 biological replicates) (**M**)

Next, we examined whether heavy metal response is involved in platinum-based chemotherapy. To do so, we generated MTF1 knockout (KO) cells using the CRISPR-Cas9 technique (Fig. 2A) and subjected them to cisplatin treatment. Although loss of MTF1 did not affect cell growth under normal culture condition (Fig. 2B), MTF1 KO cells showed enhanced sensitivity to cisplatin compared to wild-type cells (Fig. 2B-D). Specifically, the average cisplatin half-maximal inhibitory concentration (IC50) values for wild-type HEY (170 µM) and HEC1A (348 µM) are higher than those for their respective MTF1 KO cells (52 µM for HEY-MTF1 KO1, 69 µM for HEY-MTF1 KO2, 72 µM for HEY-MTF1 KO1, and 81 µM for HEY-MTF1 KO2). Additionally, cisplatin treatment further inhibited MTF1 KO HEY (Fig. 2E and F) and HEC1A (Fig. 2G and H) xenograft tumor growth compared to its inhibitory effect on wild-type tumors. This effect was specific to cisplatin, as both wild-type and MTF1 KO cells displayed similar sensitivities to other DNA damage agents, such as mitomycin C and camptothecin [29] (Fig. 2I and J). Moreover, expressing heavy metal response proteins MT1A and MT2A (Fig. 2K) significantly rescued the viability of MTF1 KO cells under cisplatin treatment (Figs. 2L and M). Specifically, although the average cisplatin IC50 values for MTF1 KO cells of HEY (19 µM) and HEC1A (28 µM) are lower than their wild-type cells (112 µM for HEY and 230 µM for HEC1A), expression of MT1A and MT2A significantly increased their IC50 values of cisplatin (38 µM for MT1A-expressing HEY-MTF1 KO1, 33 µM for MT2A-expressing HEY-MTF1 KO1, 82 µM for MT1A-expressing HEC1A-MTF1 KO1, and 104 µM for MT2A-expressing HEC1A-MTF1 KO1). Collectively, these data demonstrate the critical roles of MTF1 and its associated heavy metal response in regulating platinum-based chemotherapy.

### The Hippo pathway-mediated MTF1 regulation is crucial for platinum-based chemotherapy

**Fig. 3:**
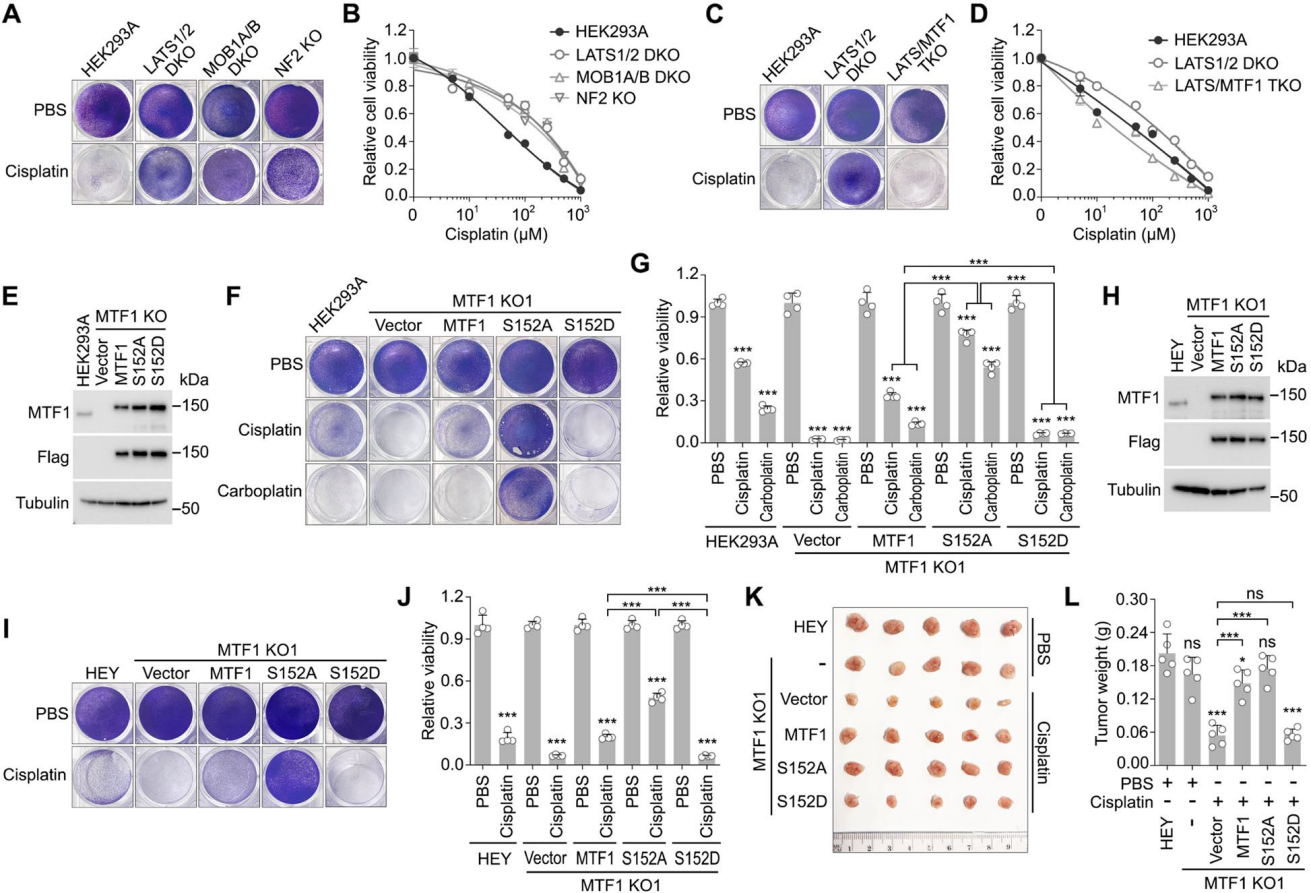
The Hippo-MTF1 pathway modulates platinum-based chemotherapy. **A-B** Loss of Hippo pathway components LATS1/2, MOB1A/B and NF2 protects cells under cisplatin treatment. The indicated Hippo pathway component knockout cells were treated with cisplatin (50 µM) for 48 h and visualized by crystal violet staining (**A**). The indicated Hippo pathway component knockout cells were treated with the indicated concentration of cisplatin for 24 h and quantified for relative viability (mean, *n* = 3 biological replicates) (**B**). **C-D** MTF1 is required for the LATS1/2 deficiency-induced cisplatin resistance. The indicated cells were treated with cisplatin (50 µM) for 48 h and visualized by crystal violet staining (**C**). The indicated cells were treated with the indicated concentration of cisplatin for 24 h and quantified for relative viability (mean, *n* = 3 biological replicates) (**D**). **E** Validation of the MTF1 KO HEK293A cells reconstituted with MTF1 and its indicated mutants. Western blot was performed using the indicated antibodies. **F-G** Reconstitution of MTF1 S152A mutant rescues the MTF1 KO HEK293A cell viability under the treatment with cisplatin and carboplatin. The MTF1KO HEK293A cells were reconstituted with MTF1 and its indicated mutants, treated with cisplatin (50 µM) and carboplatin (200 µM) for 48 h, visualized by crystal violet staining (**F**), and quantified for relative viability (mean ± s.d., *n* = 3 biological replicates) (**G**). *** *p* < 0.001 (Student’s *t*-test). **H** Validation of the MTF1 KO HEY cells reconstituted with MTF1 and its indicated mutants. Western blot was performed using the indicated antibodies. **I-J** Reconstitution of MTF1 S152A mutant rescues the MTF1 KO HEY cell viability and xenograft tumor growth under cisplatin treatment. The MTF1KO HEY cells were reconstituted with MTF1 and its indicated mutants, treated with cisplatin (100 µM) for 48 h, visualized by crystal violet staining (**I**), and quantified for relative viability (mean ± s.d., *n* = 3 biological replicates) (**J**). The MTF1 KO HEY cells were reconstituted with MTF1 and its indicated mutants, subjected to xenograft study, and treated with cisplatin (5 mg/kg) twice a week. The collected tumors at the endpoint were shown (**K**), and tumor weight was measured and quantified (mean ± s.d., *n* = 5 mice per group) (**L**). * *p* < 0.05, *** *p* < 0.001 (Student’s *t*-test). ns, no significance

We previously reported that the Hippo pathway kinase LATS1 phosphorylates MTF1 at S152, resulting in the inhibition of MTF1 and its dependent heavy metal response [25]. Here, we further examined the role of the Hippo pathway in platinum-based chemotherapy. Indeed, Hippo pathway deficiency led to increased resistance to cisplatin (Fig. 3A and B). Specifically, the average cisplatin IC50 values for Hippo pathway component KO cells (148 µM for LATS1/2 DKO, 159 µ M for MOB1A/B DKO, and 127 µM for NF2 KO) are all higher than that of wild-type HEK293A cell (44 µM). The loss of MTF1 dramatically reduced the LATS1/2 DKO cell viability under cisplatin treatment (Fig. 3C and D). Specifically, although the average cisplatin IC50 value for LATS1/2 DKO cells (105 µM) is higher than that of wild-type HEK293A cells (43 µM), MTF1 depletion largely reduced the cisplatin IC50 value for LATS1/2 DKO cells (23 µM for LATS/MTF1 TKO). These data suggest the involvement of MTF1 in Hippo pathway-mediated regulation of platinum-based chemotherapy.

To test this hypothesis, we reconstituted MTF1 KO HEK293A cells with wild-type MTF1, its S152 phosphorylation-inactive mutant S152A, and its phosphorylation-mimetic mutant S152D (Fig. 3E). These reconstituted cells were subjected to the treatment with cisplatin and carboplatin. As shown in Fig. 3F and G, restoring MTF1 S152A mutant, but not the S152D mutant, significantly rescued the viability of MTF1 KO HEK293A cells treated with cisplatin. Similar findings were observed in the ovarian cancer cell line HEY, where reconstitution with MTF1 S152A mutant, but not the S152D mutant, significantly rescued MTF1 KO HEY cell viability (Fig. 3I and J) and xenograft tumor growth (Fig. 3K and L) under cisplatin treatment. These data together show that the Hippo pathway-induced MTF1 inhibition promotes cell sensitivity to platinum-based drugs.

### Activation of the Hippo pathway enhances platinum-based chemotherapy

**Fig. 4:**
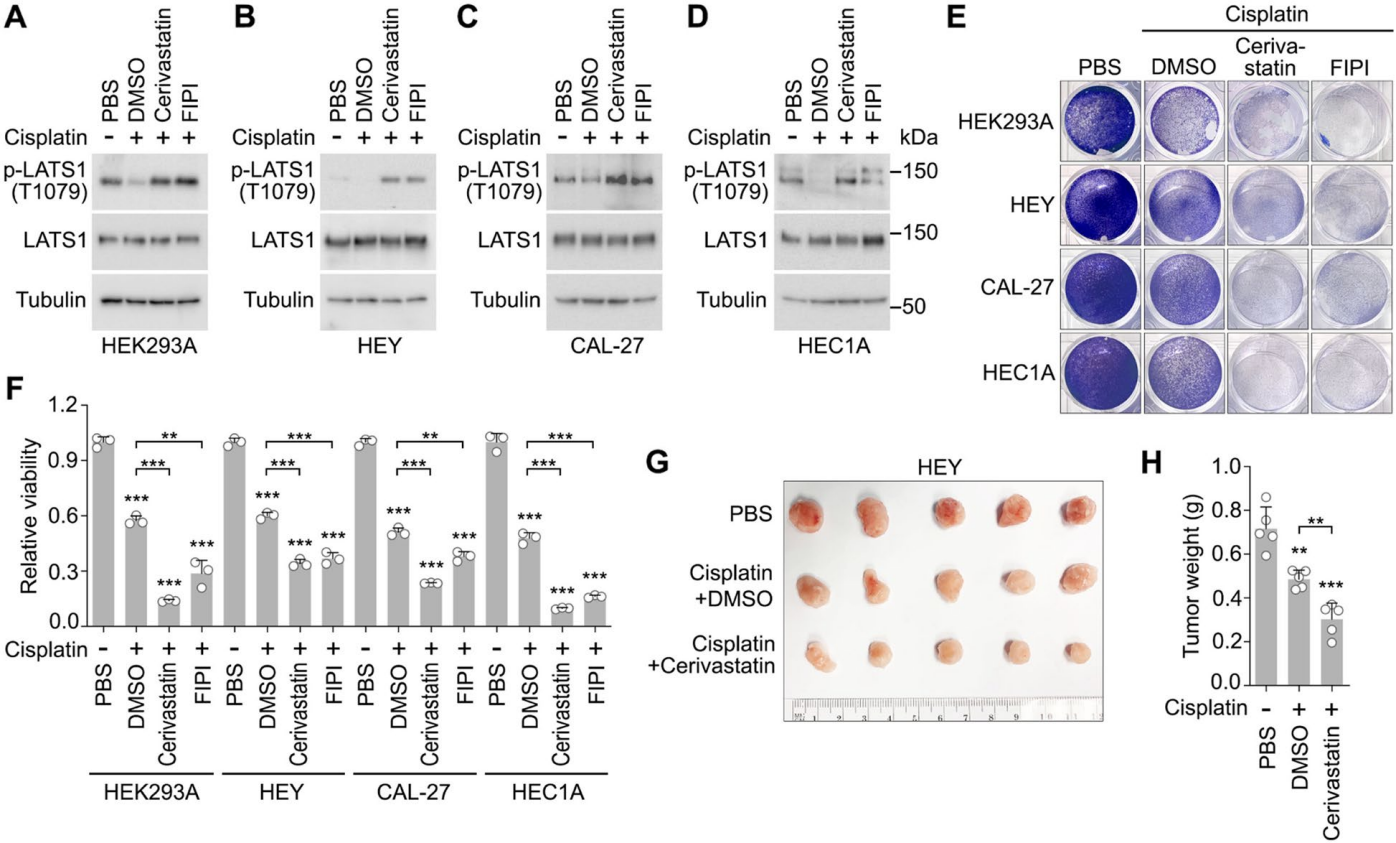
Pharmacologically activating the Hippo pathway promotes platinum-based chemotherapy. **A-D** Cerivastatin and FIPI activate LATS in the cisplatin-treated cells. The indicated cells were treated with cisplatin alone (50 µM for HEK293A, 100 µM for HEY, 10 µM for CAL-27, 100 µM for HEC1A) or with cerivastatin (10 µM) and FIPI (10 µM) for 12 h and subjected to Western blot with the indicated antibodies. (**E–F**) Combined treatment of cerivastatin and FIPI further reduces the viability of cisplatin-treated cells. The indicated cells were treated with cisplatin alone (50 µM for HEK293A, 100 µM for HEY, 10 µM for CAL-27, 100 µM for HEC1A) or with cerivastatin (10 µM) and FIPI (10 µM) for 24 h, and visualized by crystal violet staining (**E**), and quantified for relative viability (mean ± s.d., *n* = 3 biological replicates) (**F**). ** *p* < 0.01, *** *p* < 0.001 (Student’s *t*-test). (**G-H**) Treatment of cerivastatin increases the efficacy of cisplatin in targeting tumor growth. HEY xenograft tumors were treated with the indicated compounds (5 mg/kg cisplatin, 10 mg/kg cerivastatin) twice a week. The collected tumors at the endpoint were shown (**G**), and tumor weight was measured and quantified (mean ± s.d., *n* = 5 mice per group) (**H**). ** *p* < 0.01, *** *p* < 0.001 (Student’s *t*-test)

We then assessed whether combining Hippo pathway activators would benefit platinum-based chemotherapy. Like other heavy metals [25], cisplatin treatment targeted LATS activation in different cells (Fig. 4A-D). This cisplatin-induced LATS inhibition was significantly reversed by the Hippo pathway activators cerivastatin [30] and FIPI [27] (Fig. 4A-D). Moreover, treatment with cerivastatin and FIPI further reduced cancer cell viability under cisplatin treatment (Fig. 4E and F), and combined treatment with cerivastatin enhanced the efficacy of cisplatin in suppressing tumor growth (Fig. 4G and H). These findings highlight the therapeutic potential of Hippo pathway activators in improving the outcomes of platinum-based chemotherapy.

### Clinical implications of the Hippo-MTF1 pathway in platinum-based chemotherapy

**Fig. 5:**
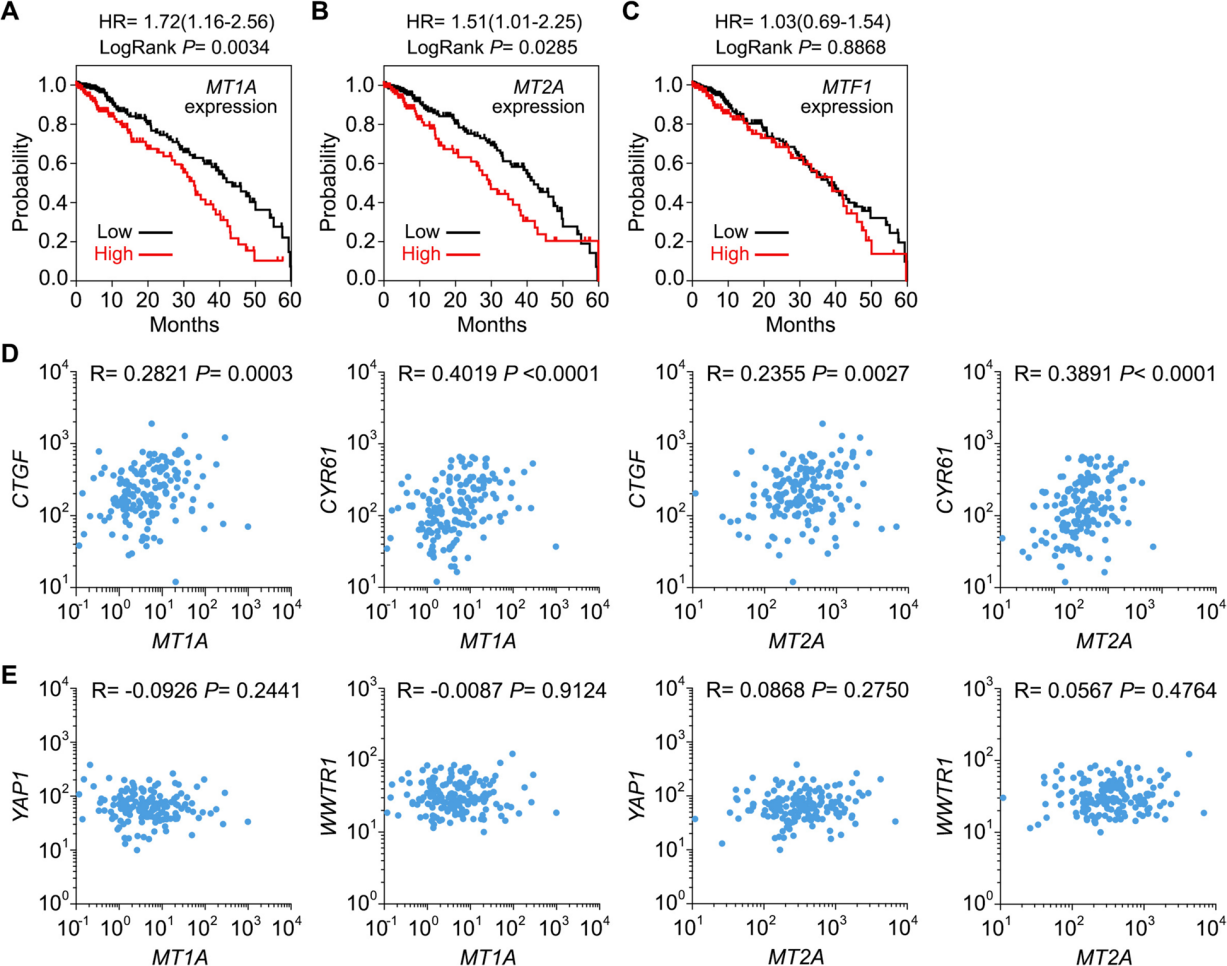
Clinical relevance of the Hippo-MTF1 pathway in platinum-based chemotherapy. **A-C** Kaplan–Meier curves of overall survival of lung adenocarcinoma (LUAD) patients is stratified by the expression levels of *MT1A* (**A**), *MT2A* (**B**) and *MTF1* (**C**). The LUAD RNAseq datasets with clinic data were downloaded from the Cancer Genome Atlas (TCGA) data portal. A total of 491 patients were analyzed for their overall survival. The *p* value was calculated by using the log-rank (Mantel-Cox) test. **D-E** The expression of heavy metal response genes *MT1A* and *MT2A* is positively correlated with that of Hippo signaling downstream genes *CTGF* and *CYR61* (**D**), but not with that of *YAP1* or *WWTR1* (**E**), in the cisplatin/carboplatin-treated LUAD patient samples. The indicated gene expression from a total of 150 patients who were treated with cisplatin and carboplatin were subjected to Spearman correction analysis. Correlation coefficient *R* value and *p* value were calculated by GraphPad prism software

To determine the clinical relevance of the Hippo-MTF1 pathway, we analyzed data from The Cancer Genome Atlas (TCGA) regarding the expression of *MTF1* and its downstream genes *MT1A* and *MT2A* in lung adenocarcinoma (LUAD) cancer patients. Notably, patients with high expression levels of *MT1A* (Fig. 5A) and *MT2A* (Fig. 5B) had poor overall survival rates, whereas MTF1 expression did not show a significant correlation (Fig. 5C). This suggests that increased transcriptional activity of MTF1 correlates with poor clinical outcomes for LUAD patients. Moreover, a positive correlation was observed between the expression of Hippo signaling downstream genes *CTGF* and *CYR61* and MTF1 downstream genes *MT1A* and *MT2A* in cisplatin/carboplatin-treated LUAD patient samples (Fig. 5D). However, no significant correlation was found between the expressions of *YAP1*/*WWTR1* and MTF1 downstream genes *MT1A* and *MT2A* in these patient samples (Fig. 5E).

To assess the broader impact of our findings, we utilized different cancer models to examine the Hippo-MTF1 pathway-mediated heavy metal response in platinum-based chemotherapy. Our data confirmed that the expression of MTF1 target genes correlates with poor overall survival in patients with breast (Figure S1A), ovarian (Figure S1B), and head and neck (Figure S1C) cancers.

Collectively, these data suggest the clinical relevance of the Hippo-MTF1 pathway in platinum-based chemotherapy.

## Discussion

Our previous work uncovered a YAP/TAZ-independent role of the Hippo pathway in control of MTF1 and its associated heavy metal response [25]. In this study, we further demonstrated the crucial role of the Hippo-MTF1 pathway in modulating platinum-based therapy, emphasizing its translational significance.

In addition to inducing DNA damage and cell cycle arrest, our data show that platinum-based compounds activate MTF1 and the heavy metal response (Fig. 1), which contributes to cellular resistance against these chemotherapy drugs (Fig. 2). Therefore, targeting MTF1 presents a promising strategy to enhance the effectiveness of platinum-based drugs. Although developing therapeutic approaches aiming at transcription factors like MTF1 has been limited and challenging [31, 32], the discovery of the Hippo pathway as a key regulator of MTF1 [25] provides a potential opportunity to achieve so. Indeed, we found that activation of the Hippo pathway inhibits MTF1, thereby sensitizing cancer cells to platinum-based chemotherapy (Fig. 3). Therefore, our study defines the role of the Hippo-MTF1 pathway in platinum-based chemotherapy, provides mechanistic insights into platinum-based chemoresistance from a new perspective, and more importantly, offers an opportunity for overcoming the limitations associated with directly targeting MTF1.

YAP and TAZ are well-known downstream effectors of the Hippo pathway, required for the Hippo pathway-associated proliferation, survival, and tissue homeostasis [13–18]. As oncogenic proteins, they drive chemoresistance to various clinical compounds, including platinum-based drugs [33]. Based on these facts, the platinum chemoresistance induced by Hippo pathway deficiency likely involves both YAP/TAZ and MTF1, albeit through distinct mechanisms. Despite this, our findings underscore the sensitizer role of the Hippo pathway in platinum-based chemotherapy by targeting both YAP/TAZ and MTF1.

The development of Hippo pathway activators is an emerging and promising area in cancer therapy [34]. While research efforts have focused on identifying small molecules to enhance the activity of the Hippo kinase LATS, progress has been slow, and specific Hippo pathway activator remain limited. Our study provides a proof-of-concept by demonstrating the sensitizing effect of the cholesterol-lowering drug cerivastatin and phospholipid D inhibitor FIPI (Fig. 4), both known to activate the Hippo pathway through different signaling mechanisms [27, 30]. These findings further underscore the potential of developing Hippo pathway activators to improve treatment outcomes and overcome resistance in cancer therapy.

## Supplementary Information

Supplementary Material 1: Figure S1 Clinical relevance of the Hippo-MTF1 pathway in breast, ovarian, and head and neck cancers. (A-B) Kaplan–Meier curves of overall survival of patients in breast (BRCA) (A) and ovarian (OV) (B) cancers is stratified by the expression levels of *MT1* and *MT2A* genes using Kaplan–Meier Plotter (https://kmplot.com/analysis/). The *p* value was calculated by using the log-rank (Mantel-Cox) test. (C) Kaplan–Meier curve of overall survival of head and neck squamous cell carcinoma (HNSC) patients is stratified by the expression levels of *MT1* and *MT2A* genes using the clinic data downloaded from the Cancer Genome Atlas (TCGA) data portal. The *p* value was calculated by using the log-rank (Mantel-Cox) test

## Data Availability

No datasets were generated or analyzed during the current study.
