# Supplementary figures and images for: The Hippo pathway promotes platinum-based chemotherapy by inhibiting MTF1-dependent heavy metal response
Source: BMC Cancer. 2025 Feb 8;25:223. doi: 10.1186/s12885-025-13661-8 (PMC11806854; doi:10.1186/s12885-025-13661-8)

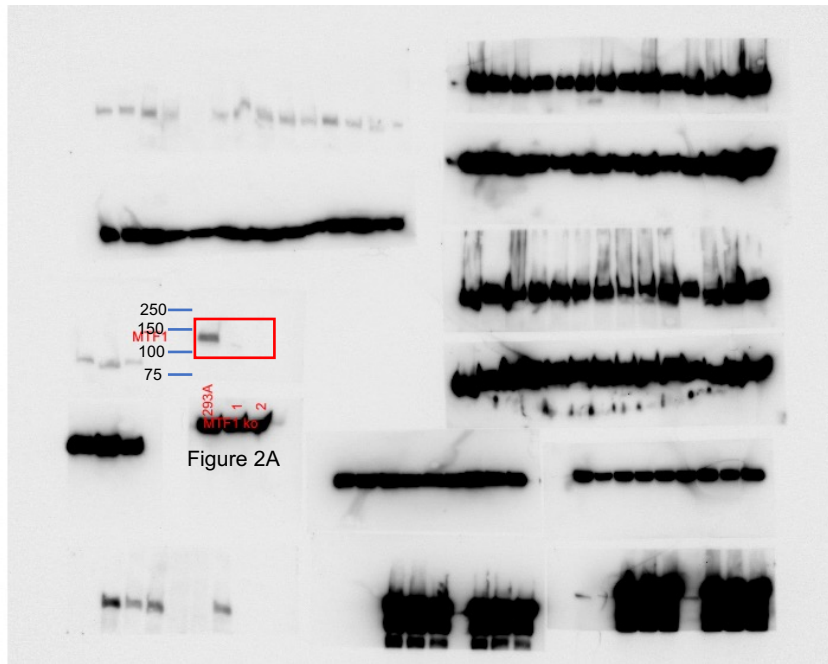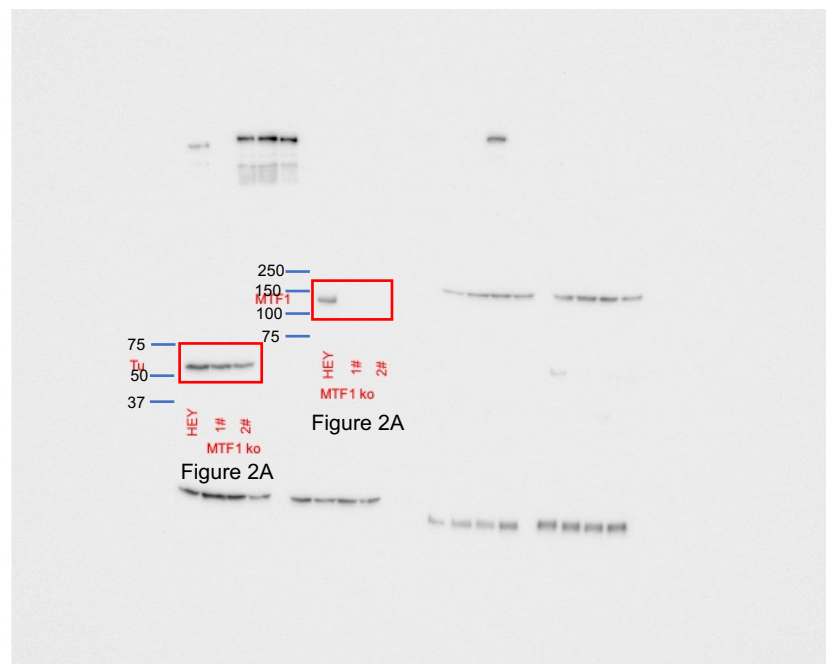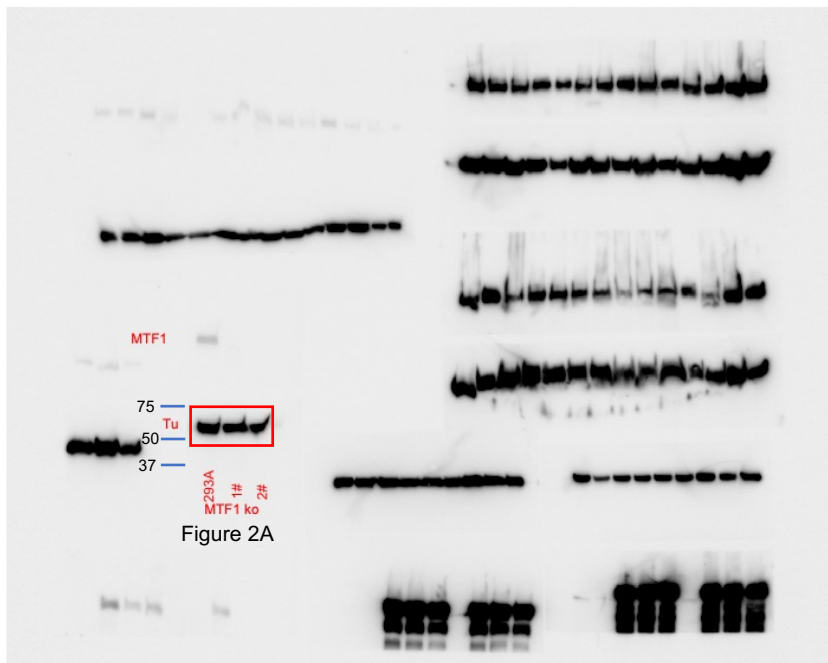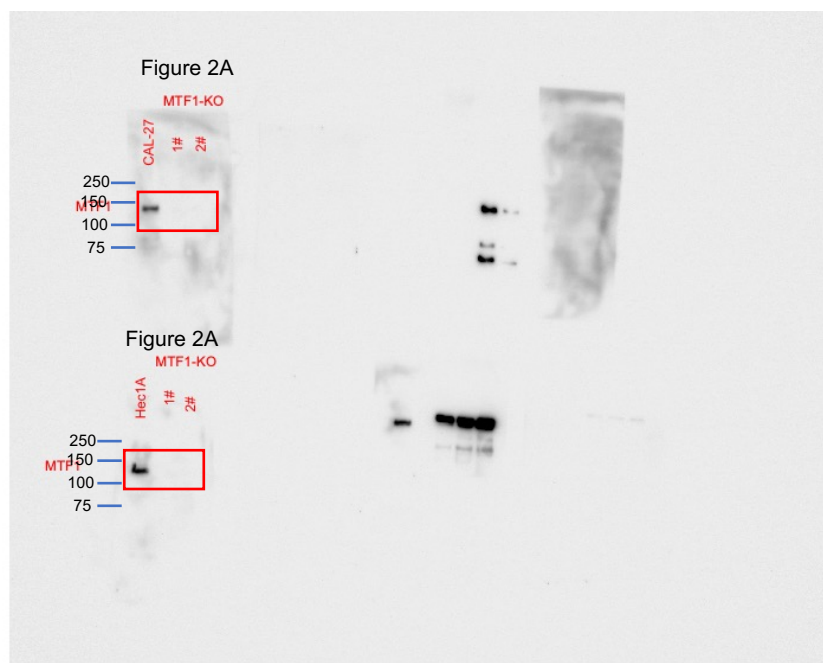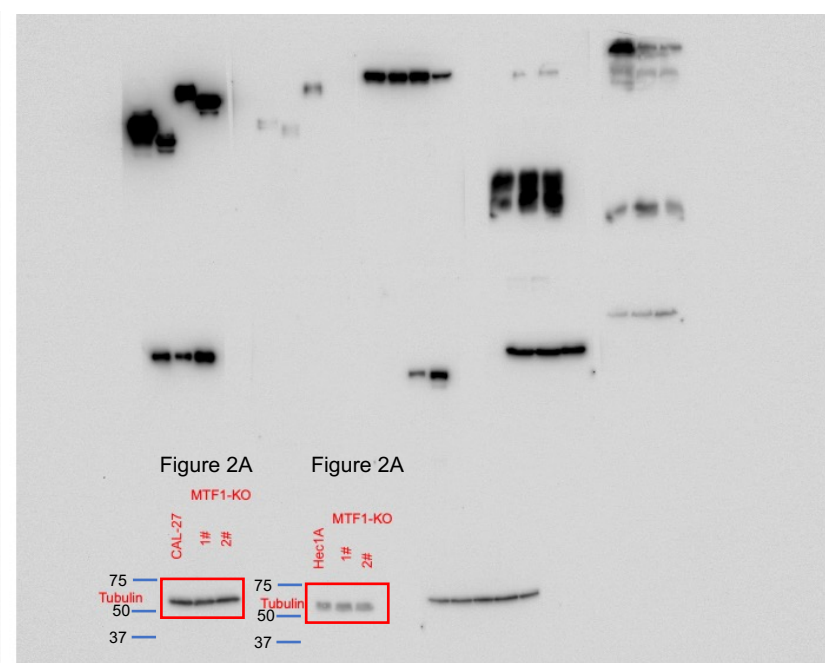

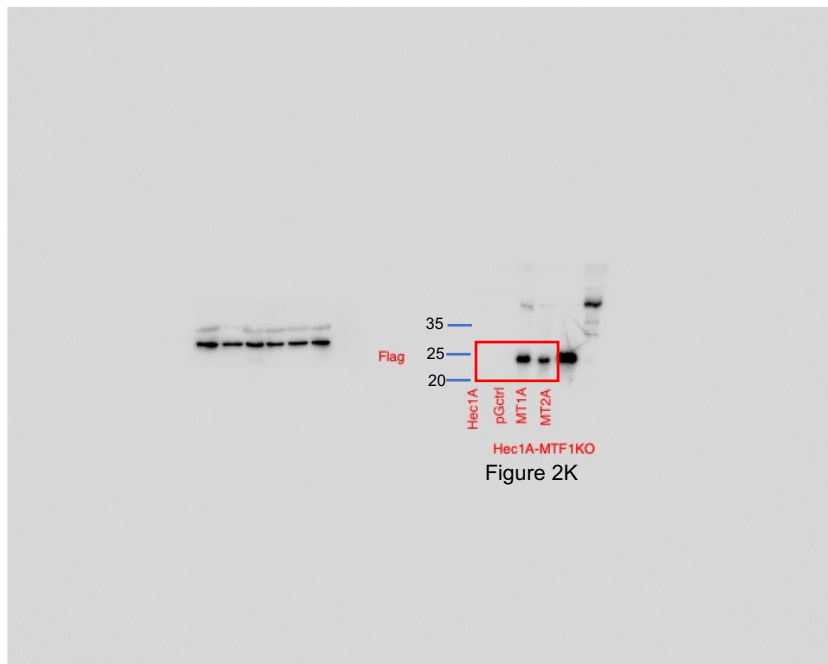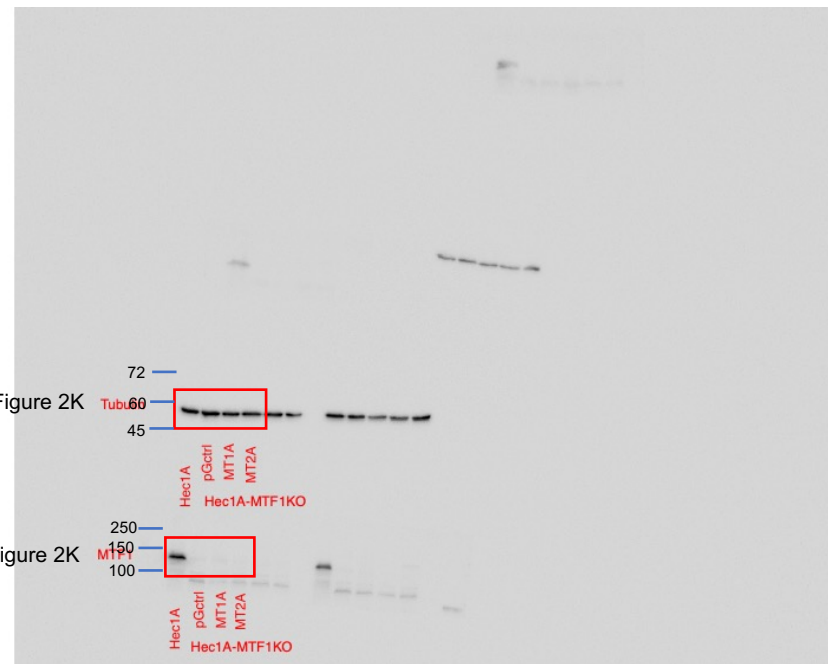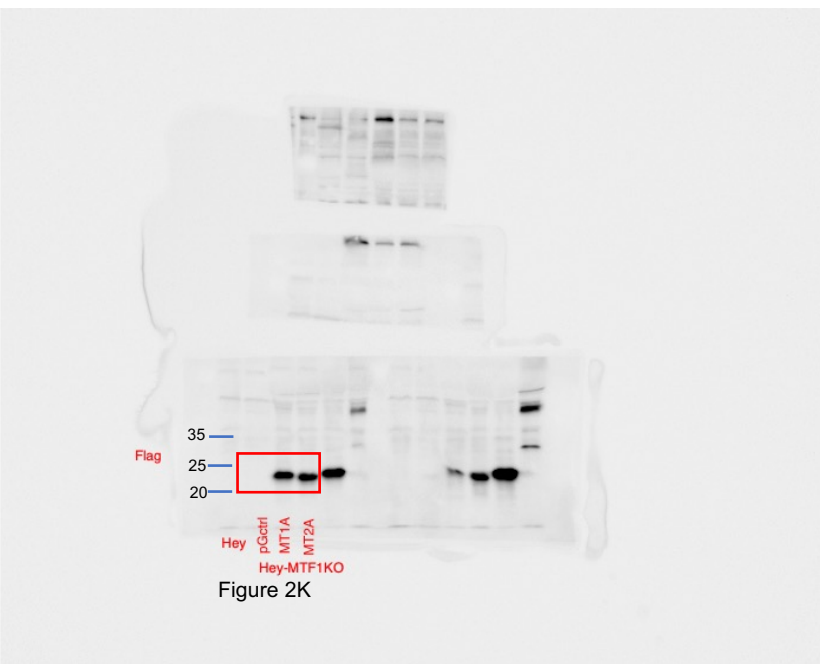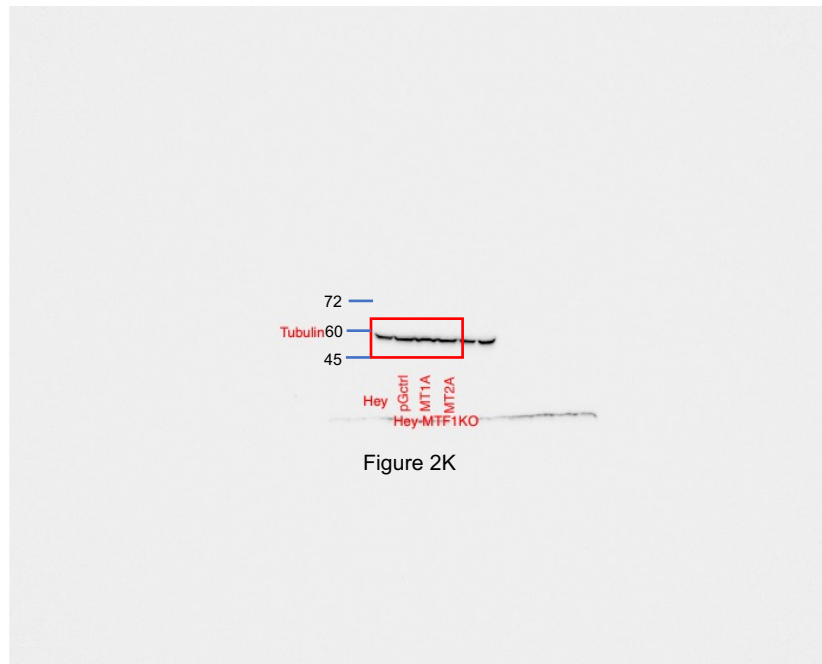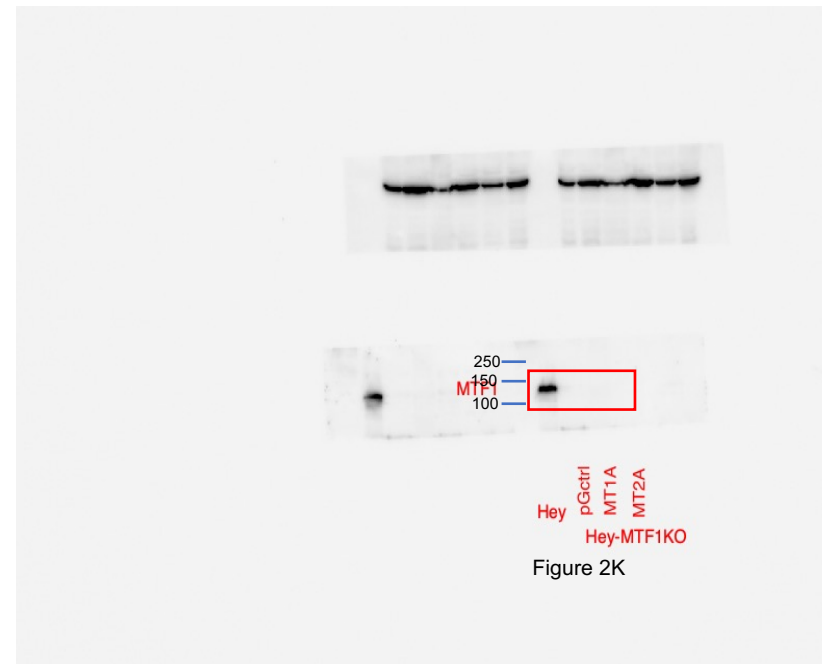

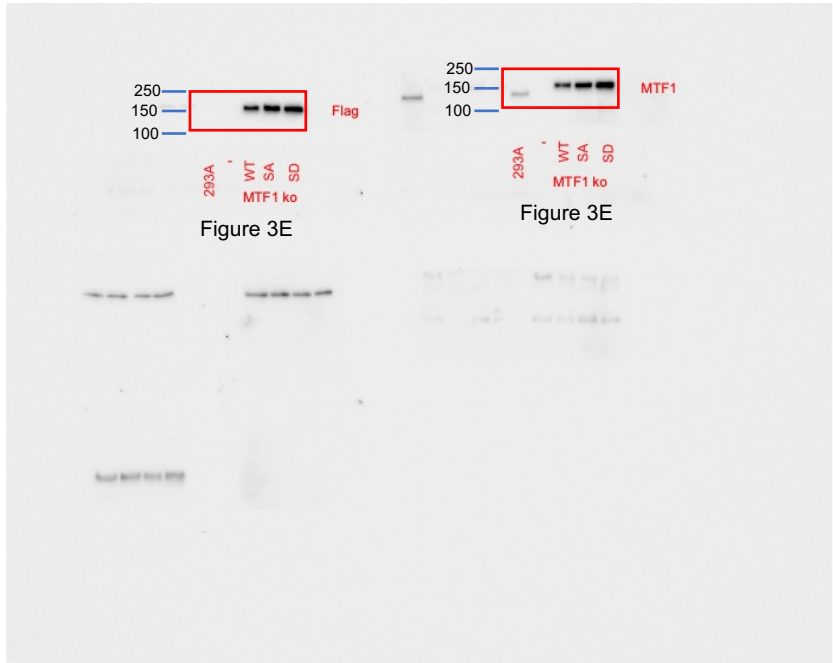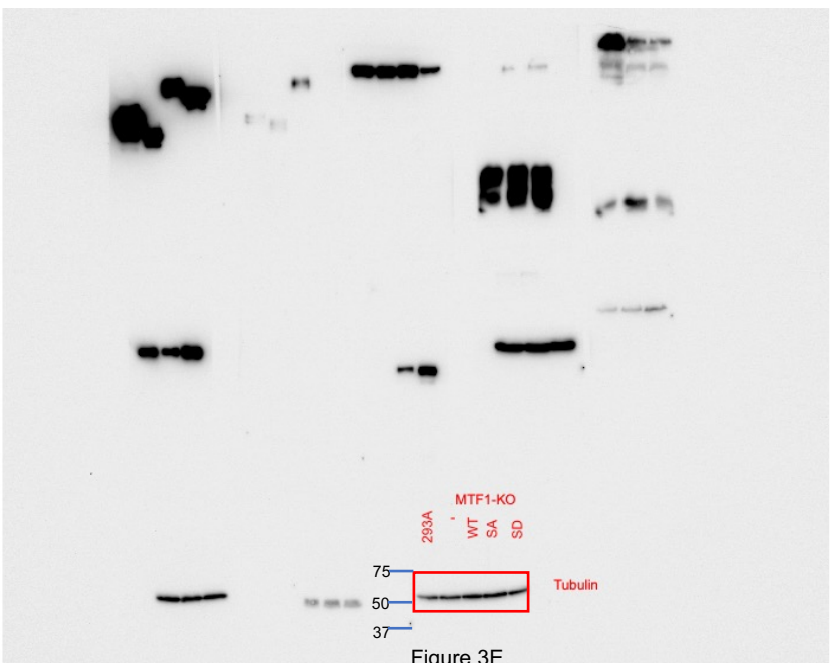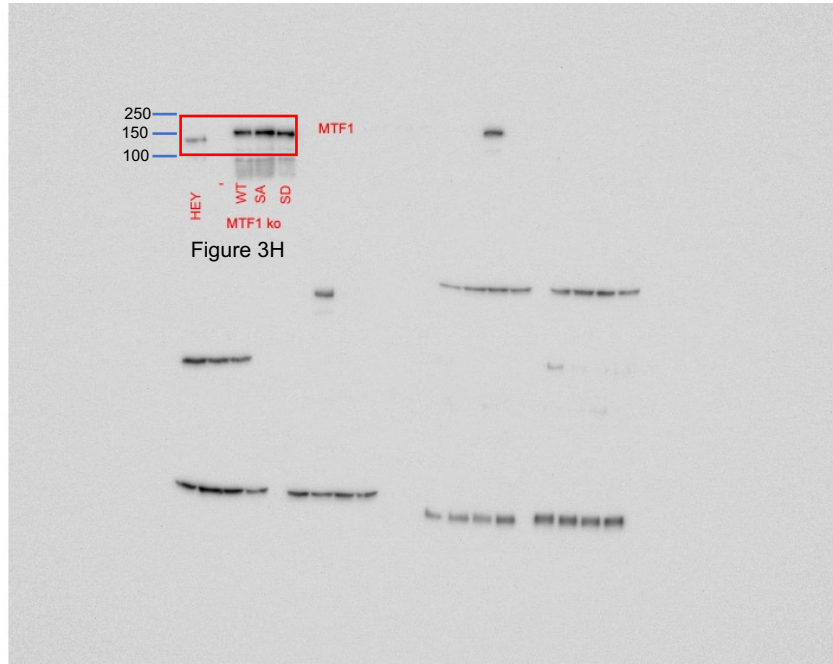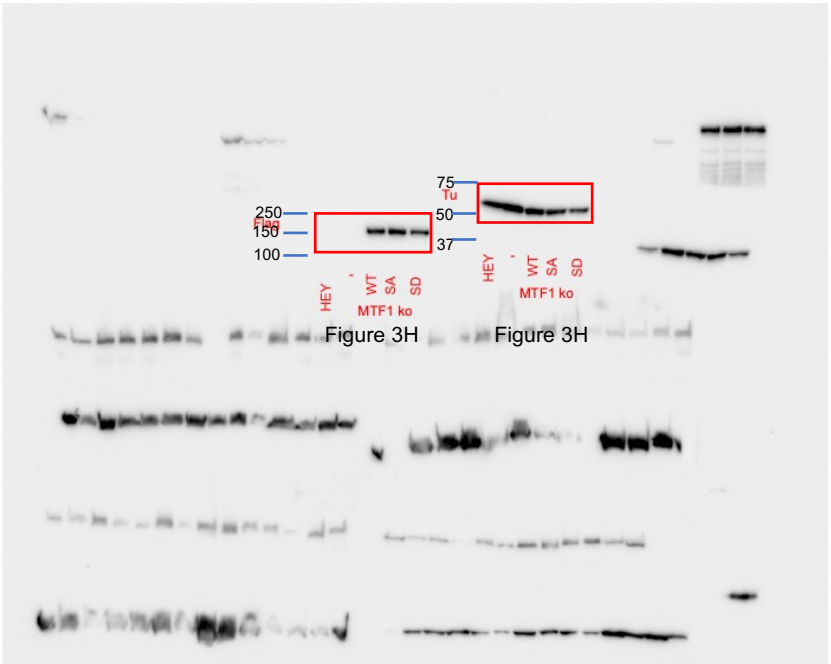

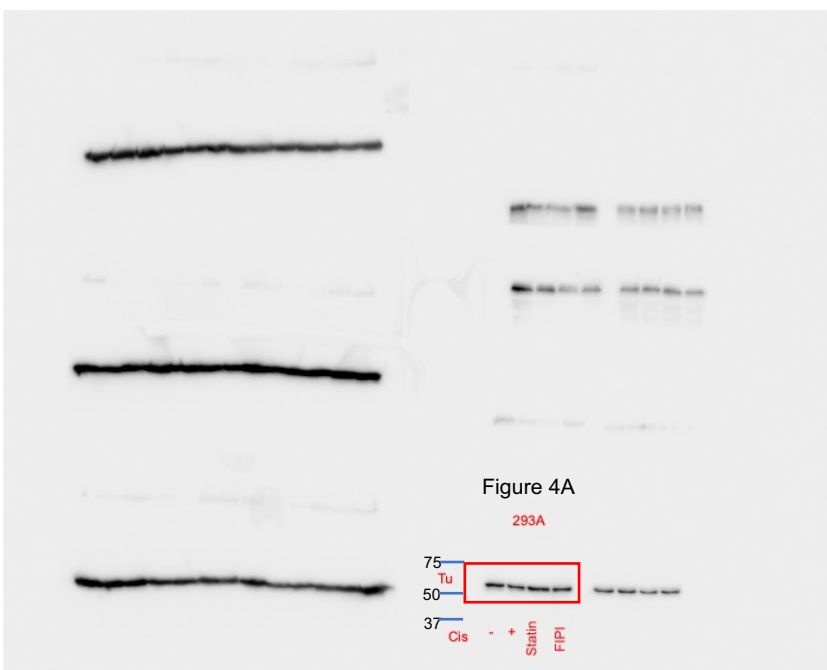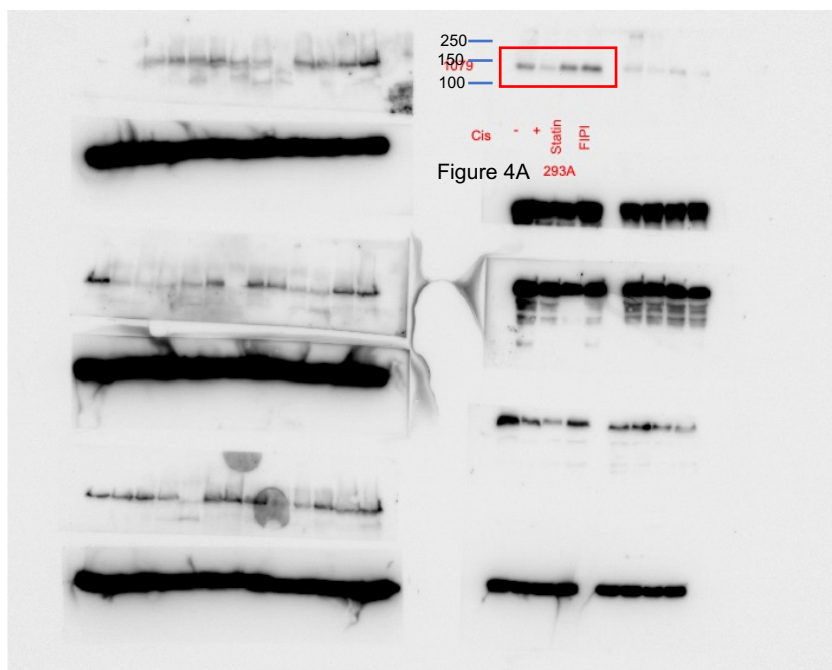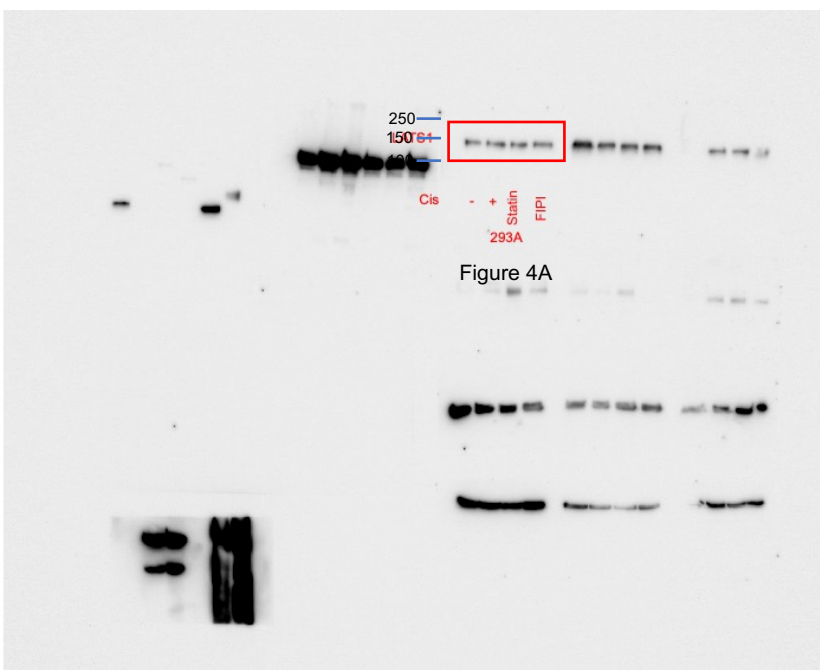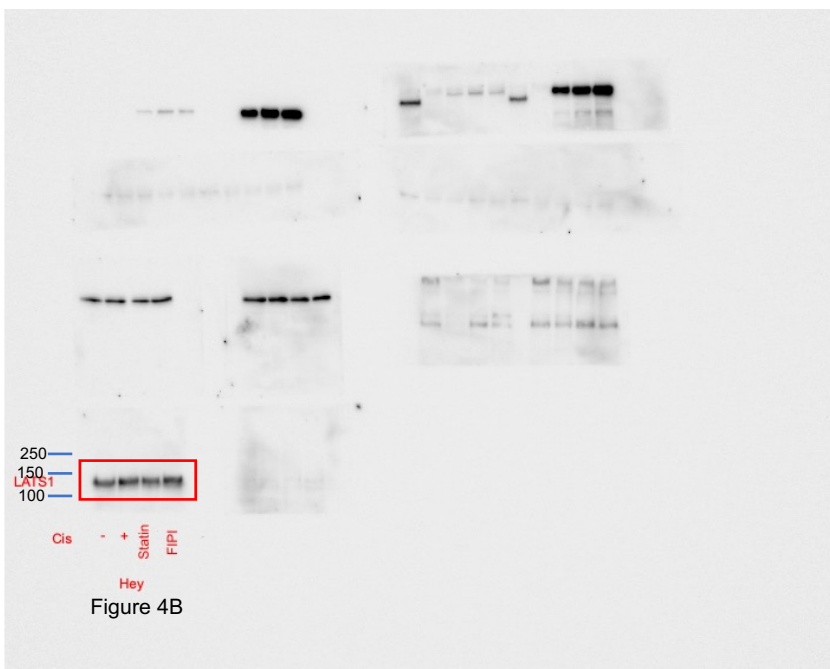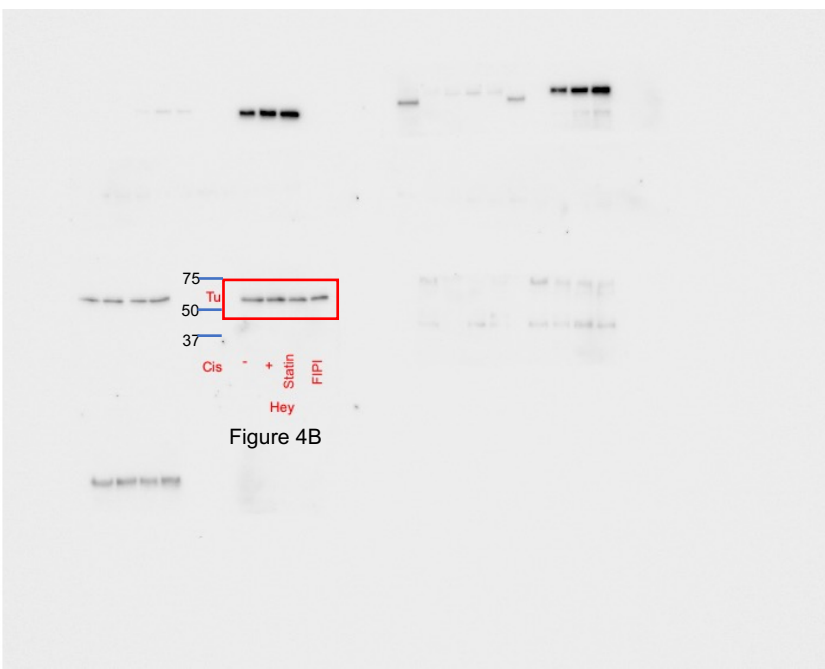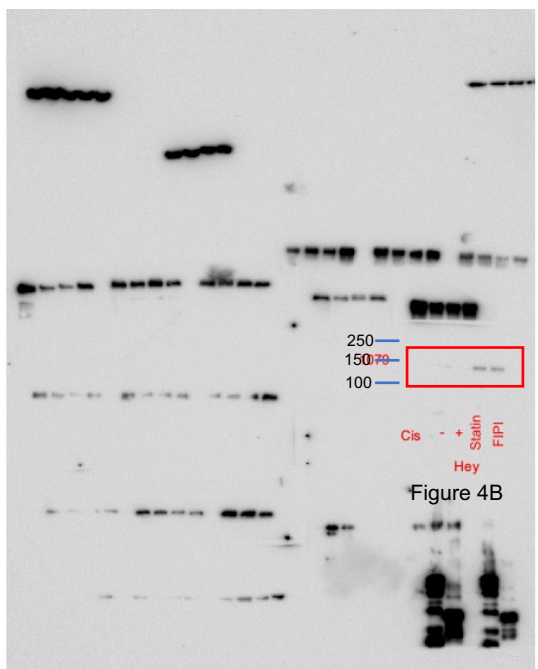

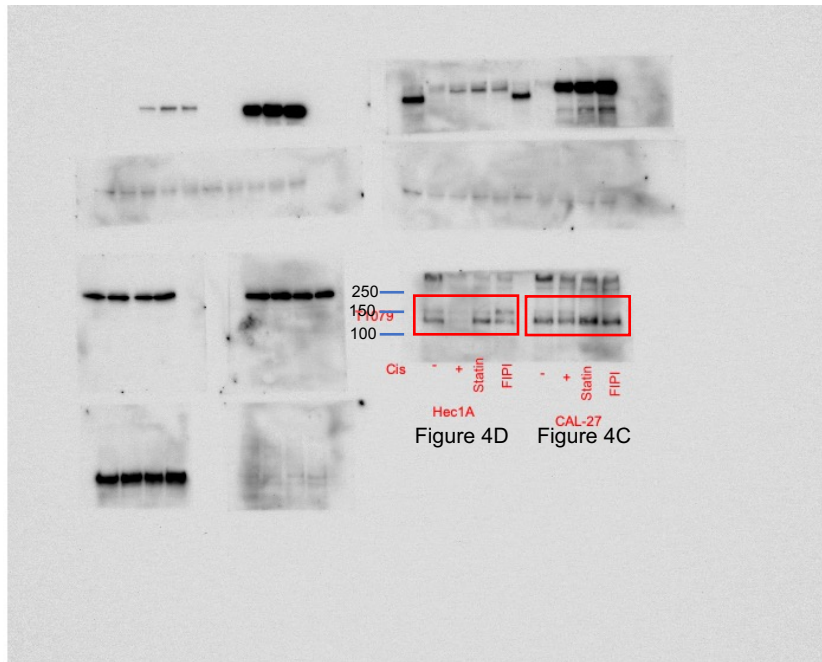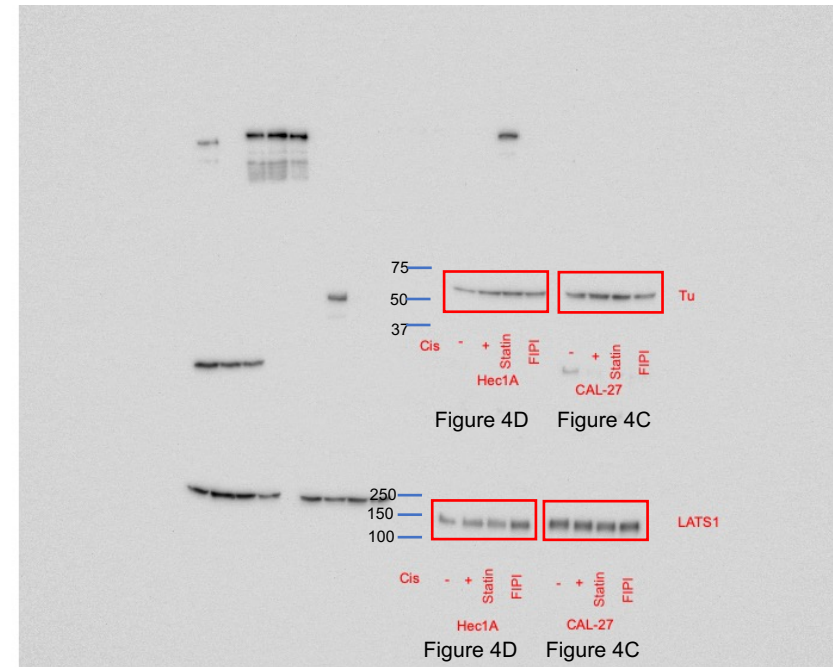

Supplement: Supplementary file 1 — Supplementary Material 1: Figure S1 Clinical relevance of the Hippo-MTF1 pathway in breast, ovarian, and head and neck cancers. (A-B) Kaplan–Meier curves of overall survival of patients in breast (BRCA) (A) and ovarian (OV) (B) cancers is stratified by the expression levels of MT1 and MT2A genes using Kaplan–Meier Plotter (https://kmplot.com/analysis/). The p value was calculated by using the log-rank (Mantel-Cox) test. (C) Kaplan–Meier curve of overall survival of head and neck squamous cell carcinoma (HNSC) patients is stratified by the expression levels of MT1 and MT2A genes using the clinic data downloaded from the Cancer Genome Atlas (TCGA) data portal. The p value was calculated by using the log-rank (Mantel-Cox) test [file 12885_2025_13661_MOESM1_ESM.pdf]
